# Automated Ischemic Stroke Classification from MRI Scans: Using a Vision Transformer Approach

**DOI:** 10.3390/jcm13082323

**Published:** 2024-04-17

**Authors:** Wafae Abbaoui, Sara Retal, Soumia Ziti, Brahim El Bhiri

**Affiliations:** 1 Intelligent Processing & Security of Systems (IPSS) Research Team, Faculty of Sciences, Mohammed V University in Rabat, Rabat 10000, Morocco; s.ziti@um5r.ac.ma; 2 SmartiLab, Moroccan School of Engineering Sciences (EMSI), Rabat 10000, Morocco; 3 2IACS Laboratory, ENSET, University Hassan II of Casablanca, Mohammedia 28830, Morocco; sara.retal@univh2c.ma

**Keywords:** deep learning, vision transformer, ischemic stroke, medical image analysis, MRI scans

## Abstract

**Background**: This study evaluates the performance of a vision transformer (ViT) model, ViT-b16, in classifying ischemic stroke cases from Moroccan MRI scans and compares it to the Visual Geometry Group 16 (VGG-16) model used in a prior study. **Methods**: A dataset of 342 MRI scans, categorized into ‘Normal’ and ’Stroke’ classes, underwent preprocessing using TensorFlow’s tf.data API. **Results**: The ViT-b16 model was trained and evaluated, yielding an impressive accuracy of 97.59%, surpassing the VGG-16 model’s 90% accuracy. **Conclusions**: This research highlights the ViT-b16 model’s superior classification capabilities for ischemic stroke diagnosis, contributing to the field of medical image analysis. By showcasing the efficacy of advanced deep learning architectures, particularly in the context of Moroccan MRI scans, this study underscores the potential for real-world clinical applications. Ultimately, our findings emphasize the importance of further exploration into AI-based diagnostic tools for improving healthcare outcomes.

## 1. Introduction

The field of healthcare has witnessed a transformative evolution in recent years, driven by the rapid advancements in Artificial Intelligence (AI) and deep learning technologies. These advancements have paved the way for innovative solutions to critical medical challenges, particularly in the domain of medical image analysis. Among these challenges, the early and accurate detection of ischemic stroke stands as a paramount concern. Ischemic stroke is a leading cause of death and disability worldwide [1,2], making its early diagnosis and prompt intervention a critical factor in patient outcomes [3,4].

Medical imaging, particularly Magnetic Resonance Imaging (MRI), has emerged as a powerful tool for diagnosing and assessing ischemic stroke [5]. The accurate interpretation of these images, however, is highly dependent on the expertise of radiologists, and it can be time-consuming, especially in a medical setting, where every second counts. The integration of AI and deep learning into this process has shown immense potential to mitigate these issues, providing automated, efficient, and consistent analyses.

To contextualize the role of vision transformers (ViTs) in this domain, it is essential to first understand the broader landscape of AI and deep learning in medical image analysis. AI, the broader field encompassing machine learning (ML) and deep learning (DL), has revolutionized medical image analysis by enabling the automated interpretation of images with high accuracy and efficiency. ML techniques enable computers to learn from data and make predictions or decisions without being explicitly programmed, while DL, a subset of ML, involves neural networks with multiple layers that can automatically learn representations of data [6]. However, classic deep learning models, such as Convolutional Neural Networks (CNNs), have limitations in capturing long-range dependencies within images.

The contributions of our study are multifaceted and impactful. Firstly, by deploying ViT-b16 in medical image analysis, we demonstrate its practical applicability in addressing critical healthcare challenges, particularly in the realm of ischemic stroke classification. This showcases the versatility of ViTs beyond traditional computer vision tasks and extends their impact to the forefront of medical diagnostics. Furthermore, our research highlights the superior performance of ViT-b16 compared to the VGG-16 model, with ViT-b16 achieving an accuracy of 97.59% compared to VGG-16’s 90%. It is noteworthy that VGG-16 was previously applied in a study on the same dataset, providing a basis for direct comparison. Moreover, our study underscores the transformative potential of advanced deep learning architectures, such as ViTs, in significantly enhancing diagnostic accuracy and efficiency. By leveraging ViT-b16, we pave the way for more precise and timely diagnoses, ultimately leading to improved patient outcomes and healthcare delivery.

The rest of this paper is organized as follows. Section 2 provides an overview of related works, contextualizing our study within existing research. In Section 3, we detail the materials and methods employed in our study. Section 4 delves into the experimental results. Following the results, Section 5 provides the discussion, covering both the main findings and study limitations. Finally, Section 6 concludes the paper by summarizing the key insights.

## 2. Related Works

Within the realm of AI for medical image analysis, ViTs have emerged as a breakthrough technology. ViTs have demonstrated exceptional performance in various computer vision tasks and exhibit the ability to capture intricate dependencies and patterns within medical images [7]. For instance, Asiri et al. [8] conducted a comprehensive study on brain tumor classification using five pre-trained ViT models, achieving a high accuracy of 98.24% with ViT-b32. Their research results surpass those of existing methodologies, demonstrating the potential of ViT models in medical image analysis and providing a benchmark for future brain tumor classification studies. Moreover, Tummala et al. [9] investigated the effectiveness of an ensemble of standard ViT models in diagnosing brain tumors from T1-weighted (T1w) MRI data. Their study demonstrated that the ensemble, comprising ViT models such as B/16, B/32, L/16, and L/32 pre-trained and fine-tuned on ImageNet, achieved an impressive overall testing accuracy of 98.7%. By leveraging the strengths of individual models and combining them, the ensemble outperformed the individual models and their ensembles at lower resolutions, highlighting its potential for the accurate computer-aided diagnosis of brain tumors based on T1w Contrast-Enhanced (CE) MRI scans. A novel approach was proposed by Cai et al. [10], who introduced the Swin Unet3D model, which innovatively combines the strengths of ViT and Convolution for the voxel segmentation of medical images. By designing a feature extraction sub-module with a parallel structure of Convolution and ViT, the model effectively learns both global and local dependency information in the image. Their proposed model achieved impressive dice coefficients on the validation datasets Brats2021 and Brats2018, highlighting its efficacy in brain tumor segmentation tasks. By striking a balance between the number of model parameters and segmentation accuracy, the Swin Unet3D model represents a significant advancement in the field of medical image segmentation. Aloraini et al. [11] further enriched this landscape by presenting a hybrid deep learning-based model for brain tumor classification. Their model ingeniously fused transformer and Convolutional Neural Networks, harnessing the strengths of both networks to improve diagnostic capabilities. Additionally, Lee et al. [12] proposed an enhanced computer-aided diagnosis algorithm optimized for brain tumor classification, achieving a performance improvement of up to 6% across four deep learning models. Their approach involved noise removal using Gaussian filters and the integration of the GridMask and Patterned-GridMask techniques, resulting in improved accuracy and F1-score, reaching 97.74% and 97.75%, respectively.

Meanwhile, Li et al. [13] ventured into the domain of Whole-Slide Image (WSI) analysis, contributing an end-to-end ViT-based deep learning architecture specifically designed for the examination of brain tumor WSIs. Their ViT-WSI model not only upheld the diagnostic performance but also offered interpretability, a vital asset for medical applications.

While ViTs have shown remarkable potential in diagnosing brain tumors, their adaptability extends to skin cancer classification as well, as evidenced by recent research efforts. Aladhadh et al. [14] have made notable strides by introducing a comprehensive framework for the classification of skin cancer. Their framework leverages dermoscopic images, and the integration of a medical vision transformer-based approach demonstrates its potential for accurate and efficient skin cancer diagnosis. Likewise, Xin et al. [15] recognized the remarkable performance of ViTs in traditional classification tasks and sought to capitalize on this strength for skin cancer diagnosis. To enhance feature extraction and reduce noise, they unveiled a novel transformer network named SkinTrans. This inventive approach holds promise for improving the precision of skin cancer classification.

Beyond dermatology and neuroimaging, ViTs are equally instrumental in addressing pneumonia and respiratory conditions, as highlighted by recent studies. Wang et al. [16] combined transformer and ResNet in the PneuNet model, demonstrating its efficacy in diagnosing COVID-19 based on Chest X-Ray (CXR) images. Okolo et al. [17] introduced the Input Enhanced Vision Transformer (IEViT), enhancing ViTs for CXR images associated with various respiratory pathologies. Additionally, Jiang et al. [18] proposed the Multisemantic Level Patch Merger Vision Transformer (MP-ViT) for the automatic diagnosis of pneumonia in CXR images, further emphasizing ViTs’ potential in respiratory healthcare.

In the context of neurodegenerative disorders, particularly Alzheimer’s disease, several research efforts have harnessed the potential of ViTs to advance disease detection and understanding. The following studies have contributed to this ongoing exploration. For example, in [19], the research proposed an attention-based mechanism that employs the ViT approach for Alzheimer’s disease detection using MRI images. In a similar vein, in [20], the research aimed to enhance the automatic detection of dementia in MRI brain data by investigating three prominent deep convolutional models (ResNet, DenseNet, and EfficientNet) along with two transformer-based architectures (MAE and DeiT) for mapping input images to clinical diagnoses. Additionally, ref. [21] utilized ViT to predict the conversion process from mild cognitive impairment to Alzheimer’s disease using structural magnetic resonance images (sMRIs). Moreover, the optimized vision transformer architecture in [22] predicts group membership by categorizing healthy adults, those with mild cognitive impairment, and Alzheimer’s patients within the same age group (>75 years) using resting-state functional (rs-fMRI) and sMRI data.

Prior research in healthcare showcases the versatility of ViTs in tasks such as brain tumor classification, skin cancer diagnosis, pneumonia detection, and Alzheimer’s disease understanding. Building on this momentum, our study focuses on the practical implementation of ViT-b16 to enhance the classification of ischemic stroke in Moroccan MRI scans. The primary goals of our research are twofold: firstly, to evaluate the performance of ViT-b16 in comparison to established models, highlighting its superior classification capabilities, and secondly, to demonstrate the efficacy of ViT-based approaches in addressing critical healthcare challenges, particularly in the context of ischemic stroke diagnosis.

The contributions of our study are significant. By showcasing the practical implementation of ViT-b16 in medical image analysis, we extend the impact of ViTs to critical areas of healthcare. Furthermore, our research underscores the potential of advanced deep learning architectures in improving diagnostic accuracy and streamlining clinical workflows.

## 3. Materials and Methods

### 3.1. Hardware and Software Setup

The deep learning model was designed and trained on a computing system powered by an Intel(R) Core(TM) i5-6300U processor, operating at a base frequency of 2.40 GHz and capable of reaching a clock speed of 2.50 GHz. This computing setup provided the necessary computational resources for training and evaluating the machine learning model.

The development and training of the model were carried out within a Jupyter Notebook environment. The Jupyter Notebook offers an interactive and versatile platform for model development, allowing for seamless experimentation and code iteration.

To support the implementation of the deep learning model, a range of Python libraries and dependencies played a pivotal role. These libraries, including but not limited to TensorFlow, NumPy, Pandas, Matplotlib, and Scikit-Learn, were instrumental in data preprocessing, model construction, performance evaluation, and visualization.

### 3.2. Data Collection

The dataset used in this study, obtained from the Mohammed VI University of Sciences and Health (UM6SS), comprises a total of 342 Moroccan MRI scans specifically related to cases of ischemic stroke. These scans are categorized into two classes: ’Normal’ and ’Stroke’. The dataset exhibits a balanced gender distribution, with 189 (55%) female patients and 153 (45%) male patients.

Each MRI scan in our dataset consists of 2D images obtained from individual patients, with an average of approximately 15 MRI slices per patient for analysis. The MRI sequences used in this study primarily consisted of Axial T2 FLAIR sequences, which are known for their effectiveness in detecting stroke-related lesions due to the high contrast they provide between the cerebrospinal fluid and brain tissue. This contrast enhances the visibility of abnormalities such as edema and ischemic lesions.

To organize and structure the dataset for analysis, we created Pandas DataFrames. These DataFrames contain two key columns: ’image_path’, which provides the file paths to the MRI scan images, and ’label’, which indicates the corresponding class labels. To facilitate the machine learning process, we performed label encoding, transforming the class labels into ’label_encoded’ numerical values.

**Figure 1:**
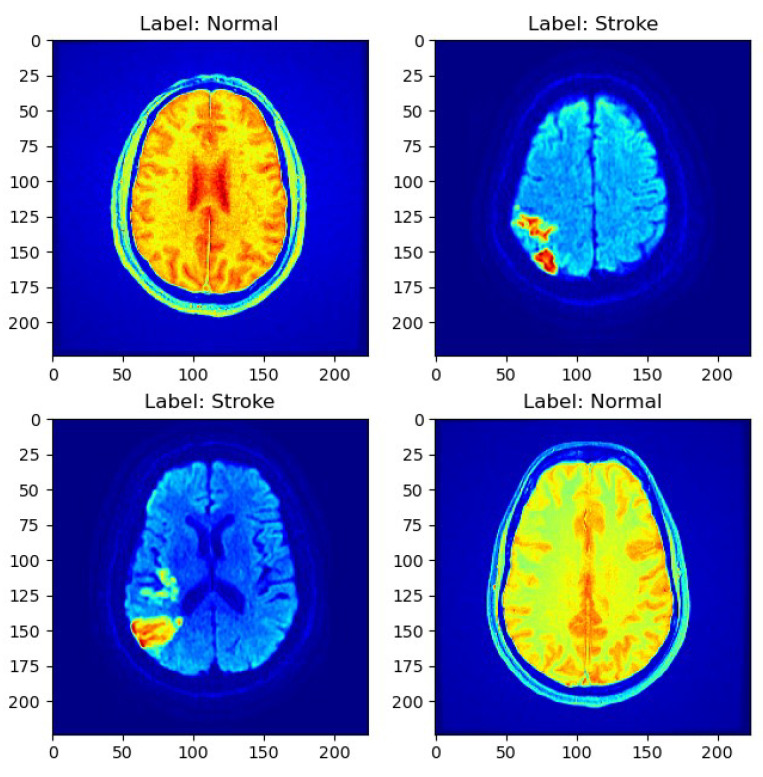
Sample MRI scans.

As depicted in Figure 1, a sample MRI scan exemplifies the diversity of cases present in the dataset, encompassing both normal and stroke cases.

### 3.3. Data Preprocessing

In preparation for model training and evaluation, we meticulously preprocessed the MRI scan images and established a structured input data pipeline. The original size of the MRI scan images varied, with the vast majority having sizes of either 512 × 512 or 256 × 256 pixels. For compatibility with the ViT model, the images were resized to 224 × 224 pixels before being fed into the model for training and evaluation. This resizing ensured uniformity in input dimensions and facilitated the integration of the images into the ViT architecture, enabling consistent processing across the dataset.

**Figure 2:**
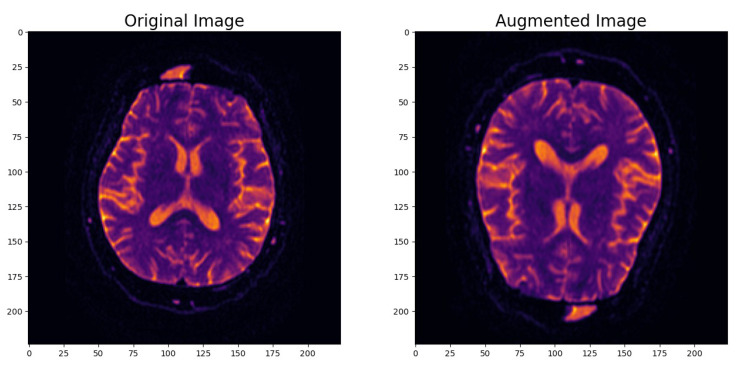
Example of augmented image.

The data preprocessing steps were as follows:

- *Data Splitting*: To ensure clear demarcation for training and evaluation, the dataset was initially partitioned into three primary subsets: a training set, a validation set, and a test set. The training set encompassed 205 samples, while the validation and test sets were amalgamated, totaling 137 samples. This partitioning strategy effectively allocated data for model training, validation, and the ultimate testing phase. Additionally, the preprocessing phase involved the further segregation of the combined validation and test set into two distinct subsets: a dedicated validation set and a test set. This strategic separation guaranteed the availability of exclusive data for model evaluation and final testing, thereby reinforcing the trustworthiness of our research findings. The validation set contained 60% of the data, facilitating model performance assessment and fine-tuning during training, while the remaining 40% comprised the test set, furnishing an independent dataset for the conclusive evaluation of the model’s classification performance.
- *Image Data Augmentation*: To enhance the model’s robustness and its ability to generalize effectively, we introduced an image data augmentation layer. This layer applied random horizontal and vertical flips as well as zooming to the images. The augmentation process simulated real-world variations in MRI scans and introduced variability into the dataset. As shown in Figure 2, this data augmentation process effectively transformed the original images, enhancing the dataset’s diversity and introducing the necessary variability for robust model training.
- *Input Data Pipeline with the tf.data API*: To create a streamlined and efficient input data pipeline, we leveraged the TensorFlow ‘tf.data’ API. Table 1 provides an overview of the key components within the input data pipeline, emphasizing their roles in optimizing data processing for model training and evaluation.

### 3.4. Model Training and Evaluation

The vision transformer model employed in this study, which comprises transformer encoder blocks encompassing Layer Normalization, Multi-head Attention, and Multi-Layer Perceptrons (MLPs), effectively captures dependencies within the images. In this research, the pre-trained ViT-b16 model formed the cornerstone of our vision transformer.

The architecture of the vision transformer model, identified as ‘vit_b16_sequential_model’, was precisely defined, as detailed in Table 2. The ’Param #’ column in Table 2 represents the number of trainable parameters in each layer of the model. These parameters include weights and biases, which are adjusted during the training process to minimize the loss function. Following the model definition, we initiated the training process. This involved compiling the model using a categorical cross-entropy loss function, with the Adam optimizer set at a learning rate of 0.001 while monitoring the accuracy metric. The training spanned 50 epochs, culminating in an impressive training accuracy of 97.56%.

In optimizing the ViT model’s performance, we carefully tuned several key hyperparameters. Specifically, we set the learning rate to 0.001 and employed a batch size of 32 during training. Our training regimen spanned 50 epochs, ensuring comprehensive learning. To mitigate overfitting, we applied a dropout rate of 0.2. The activation function used was softmax, while the loss function employed was categorical cross-entropy. This tuning process aimed to enhance the model’s accuracy and reliability in classifying ischemic strokes.

Subsequently, we evaluated the model’s performance on the test dataset, which yielded an evaluation accuracy of 97.59%. In addition, we generated model predictions and computed a classification report, revealing an accuracy of 98%. These results collectively underscore the model’s proficiency in accurately classifying ischemic stroke cases from Moroccan MRI scans.

To augment our understanding of the model’s inner workings, we delved into the architecture of ViT, a state-of-the-art deep learning approach tailored for computer vision tasks. ViT models diverge from conventional CNNs and instead leverage the Transformer architecture, originally designed for natural language processing.

**Figure 3:**
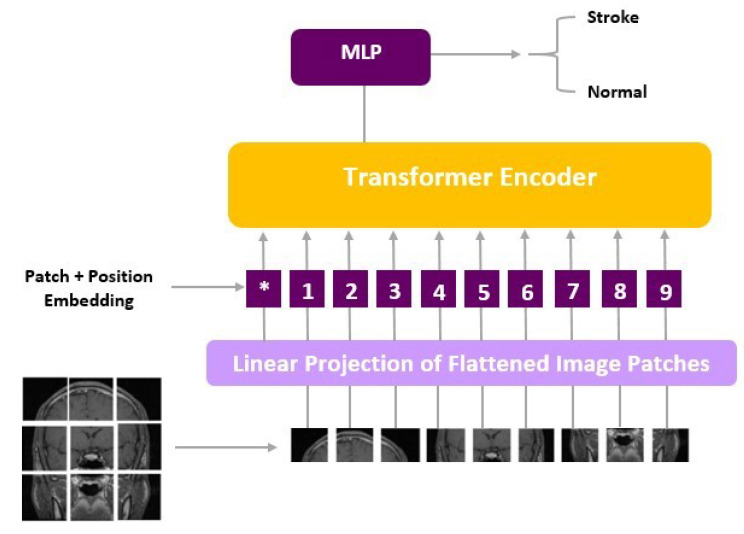
ViT architecture.

**Figure 4:**
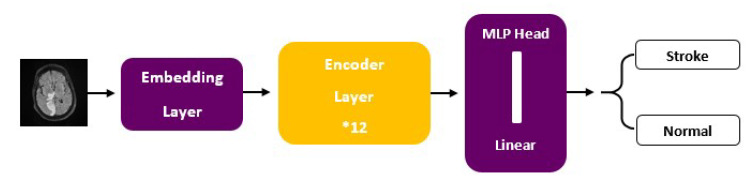
ViT-b16 architecture.

ViT models, including ViT-b16, incorporate distinct components, such as an Embedding Layer, a stack of Encoder Layers (12), the MLP Head Linear, and the final classification layer that distinguishes between ‘Stroke’ and ‘Normal’ cases. Notably, ViT-b16, the variant we employed, features 16 attention heads, establishing a robust track record in various computer vision applications. For a visual depiction of these architectural elements, please refer to Figure 3 (ViT) and Figure 4 (ViT-b16).

## 4. Results

In this section, we delve into the experimental results obtained from the ViT-b16 model applied to the task of ischemic stroke classification from Moroccan MRI scans. To evaluate the model’s performance, we used the confusion matrix and the classification report, providing a comprehensive assessment of its classification capabilities.

### 4.1. Confusion Matrix

**Figure 5:**
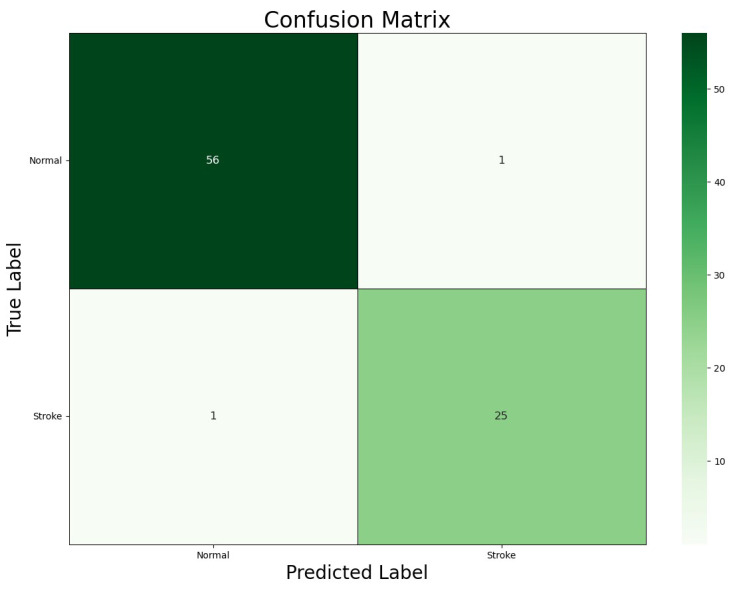
Confusion matrix for the ViT-b16 model.

Figure 5 illustrates the confusion matrix generated by our ViT-b16 model, a vital tool for assessing classification accuracy in healthcare applications. Each cell in the matrix represents a combination of predicted and actual class labels, including True Positives (TPs), True Negatives (TNs), False Positives (FPs), and False Negatives (FNs), providing valuable insights into the model’s performance.

The confusion matrix revealed that the ViT-b16 model achieved 25 TPs and 56 TNs, indicating accurate classifications of ‘Stroke’ and ‘Normal’ cases, respectively, with only 1 FP and 1 FN, representing minimal misclassifications. These findings underscore the model’s high accuracy in distinguishing between ‘Stroke’ and ‘Normal’ classes, which is crucial for clinical decision-making in stroke diagnosis. In summary, the confusion matrix offers a comprehensive overview of the model’s performance, highlighting its suitability for real-world applications.

### 4.2. Classification Report: Model Performance Metrics

In order to comprehensively assess the performance of our model, we utilized a classification report that presents various performance metrics. These metrics offer insights into the model’s precision, recall, *F*1-score, and support for each class.

- *Precision (P)*: Precision measures the ability of the model to correctly identify positive cases out of the total cases predicted as positive. It is calculated as follows:
  (1) $$P=\frac{TP}{TP+FP}$$
- *Recall (R)*: Recall, also known as sensitivity or the True Positive rate, represents the model’s ability to identify all relevant instances in the dataset. It is calculated as follows:
  (2) $$R=\frac{TP}{TP+FN}$$
- *F*1*-Score (F*1*)*: The *F*1-score is the harmonic mean of precision and recall, providing a balanced measure of the model’s performance. It is calculated as follows:
  (3) $$F1=\frac{2*P*R}{P+R}$$
- *Support*: Support refers to the number of samples in the dataset that belong to a specific class.

These metrics are calculated individually for each class in the dataset, allowing us to evaluate the model’s performance on a per-class basis. The classification report (Table 3) provides a comprehensive summary of these metrics for both the ‘Normal’ and ’Stroke’ classes, along with macro and weighted averages computed across all classes.

The ‘Macro Avg’ represents the unweighted average of precision, recall, and F1-score across all classes. The ‘Weighted Avg’ accounts for class imbalances, providing a weighted average based on the number of samples in each class.

These metrics offer a comprehensive assessment of the ViT model’s performance and enable a direct comparison with the accuracy achieved by our previous VGG-16 model, as reported in our prior study.

## 5. Discussion

The ViT-b16 model demonstrated exceptional performance in classifying ischemic stroke cases from Moroccan MRI scans, achieving an impressive accuracy of 97.59% on the evaluation dataset. This result surpasses the accuracy obtained in a previous study that utilized the VGG-16 model on the same dataset [23]. For a comprehensive comparison, we present the accuracy metrics of all models, including ViT-b16 and the baseline models (VGG-16, ResNet50, InceptionV3, and VGG-19), in Table 4 below.

It should be noted that the ResNet50, InceptionV3, and VGG-19 models were used in the previous study, which employed the VGG-16 model as the baseline. This comparison highlights the superior performance of the ViT-b16 model not only against VGG-16 but also against other established models commonly used in medical image analysis.

The utilization of ViT-b16 in this study represents a novel and innovative approach to enhancing the classification of ischemic stroke in MRI scans. By leveraging advanced deep learning architectures, we have demonstrated the potential of ViTs in addressing critical healthcare challenges. Furthermore, our research contributes to the growing body of literature on the application of ViTs in medical imaging, extending their impact to real-world clinical settings.

These findings underscore the importance of integrating cutting-edge technologies like ViTs into medical image analysis workflows. The exceptional performance of the ViT-b16 model highlights its efficacy in improving diagnostic accuracy and streamlining clinical workflows. Moving forward, further research and exploration in this area will continue to advance the field of medical image analysis, ultimately benefiting patient care and outcomes.

### Study Limitations

Our study has highlighted the potential of ViT models for classifying ischemic stroke cases from MRI scans. However, several avenues for future research exist. Firstly, despite achieving impressive accuracy, the study’s reliance on a relatively small dataset limits generalizability. Future work will focus on expanding the dataset to include a larger and more diverse set of MRI scans. Secondly, unique challenges in ischemic stroke classification, such as variations in image quality and lesion characteristics, require specialized adaptations of the ViT model. Future research will investigate domain-specific approaches to improving classification accuracy. Thirdly, the validation of the model in clinical settings and the assessment of its impact on patient care are essential. Future studies will focus on integrating the ViT-b16 model into existing clinical workflows and conducting prospective studies to evaluate its diagnostic accuracy. Lastly, beyond ischemic stroke classification, there is potential to explore the application of ViT models in other areas of medical imaging and healthcare. This includes investigating their use in detecting other neurological disorders, pathology detection, and personalized medicine. In summary, our study sets the stage for further advancements in medical image analysis, aiming to contribute to the development of more accurate and clinically relevant AI-based solutions for improving healthcare outcomes.

## 6. Conclusions

In conclusion, our study has demonstrated the efficacy of the ViT model, specifically the ViT-b16 variant, in diagnosing ischemic stroke cases from Moroccan MRI scans. With an impressive accuracy of 97.59% on the evaluation dataset, the ViT-b16 model has surpassed the performance of our prior VGG-16 model, which achieved 90% accuracy on the same dataset. This outcome underscores the ViT model’s superiority in handling complex image classification tasks.

The application of deep learning and AI, as showcased by our research, offers promising opportunities for enhancing medical image analysis. With its ability to capture intricate dependencies and patterns within medical images, the ViT model holds significant potential for real-world clinical applications. By leveraging this advanced technology, we can advance diagnostic accuracy, streamline healthcare processes, and ultimately improve patient outcomes.

As AI and deep learning continue to evolve, it is essential to further explore the potential of these models in various healthcare domains. Our research serves as a stepping stone, highlighting the ViT-b16 model’s strengths in medical imaging. We hope this study inspires future investigations and collaborative efforts to harness the power of AI for the betterment of healthcare and medical diagnosis.

## Summary Table

This study makes the following contributions:

- ViT-b16’s performance in classifying ischemic stroke cases from Moroccan MRI scans is evaluated.
- ViT-b16’s performance is compared with that of the VGG-16 model.
- ViT-b16’s practical applicability in medical image analysis is demonstrated.
- The versatility of ViTs beyond traditional computer vision tasks is highlighted.
- ViT-b16’s superior performance, with an accuracy of 97.59%, is underlined.
- The transformative potential of advanced deep learning architectures is underscored.
- The results pave the way for more precise and timely diagnoses in medical settings.

## Figures and Tables

**Table 1:** The components of the input data pipeline.

Pipeline Component	Description
Load Images	Load images from their respective file paths
Data Augmentation	Apply data augmentation if specified, enhancing model robustness
Shuffle Data	Shuffle data for a more robust training process
Batch Data	Batch data for simultaneous processing, optimizing training
Cache Data (Optional)	Optionally cache data to improve data access speed
Prefetch Data	Prefetch data for accelerated training (memory trade-offs)

**Table 2:** Model architecture summary.

Layer (Type)	Output Shape	Param #
vit-b16 (Functional)	(None, 768)	85798656
dropout (Dropout)	(None, 768)	0
dense (Dense)	(None, 512)	393728
dense_1 (Dense)	(None, 256)	131328
dense_2 (Dense)	(None, 24)	6168

**Table 3:** Classification report.

	Precision	Recall	F1-Score	Support
Normal	0.98	0.98	0.98	57
Stroke	0.96	0.96	0.96	26
Accuracy			0.98	83
Macro Avg	0.97	0.97	0.97	83
Weighted Avg	0.98	0.98	0.98	83

**Table 4:** Accuracy comparison of various models in ischemic stroke classification from Moroccan MRI scans.

Model	Accuracy
ViT-b16	97.59%
VGG-16 [23]	90%
ResNet50 [23]	87%
InceptionV3 [23]	82%
VGG-19 [23]	81%

## Data Availability

The datasets generated and analyzed during the current study are available from the corresponding author on reasonable request.
